# The Frequency and Content of Prenatal Care Determine Birth Place: A Community Based Case-Control Study

**Published:** 2019-01

**Authors:** Can ONER, Binali CATAK

**Affiliations:** 1. Department of Family Medicine, Kartal Dr Lütfi Kırdar Education and Research Hospital, Istanbul, Turkey; 2. Department of Public Health, School of Medicine, Kafkas University, Kars, Turkey

**Keywords:** Prenatal care, Home delivery, Homebirth, Institutional delivery, Birthplace

## Abstract

**Background:**

High-quality prenatal care services could decrease maternal-neonatal mortality and morbidities. The aim of this study was to compare institutional and unplanned home deliveries with regard to the use and content of prenatal care services.

**Methods:**

The study was conducted in 2011 with two hundred and twenty-nine mothers with unplanned deliveries at home as study group and 458 mothers having institutional deliveries as controls living in Istanbul. The content of prenatal care was evaluated in accordance with the national ministry of health prenatal care management guidelines and the data was collected by a questionnaire with face to face interview.

**Results:**

Women with unplanned deliveries at home use prenatal care services less frequently. Percentage of mothers that had home deliveries and did not use prenatal care services was 16.2% while this was only 3.4% in women having institutional deliveries (*P*=0.001). Moreover, all parameters of prenatal care were poor in content compared to women with institutional deliveries.

**Conclusion:**

Adequate prenatal care services in terms of quantity and quality can promote institutional deliveries.

## Introduction

Maternal mortality is one of the most important public health problems throughout the world, especially in developing and underdeveloped countries. Skilled attendance at delivery is the most crucial factor in reducing maternal-neonatal mortality and morbidities. Increasing skilled health personnel attending births is within the aims of Millennium Development Goal (1). In Turkey, a dramatic decrease has been in the number of unplanned deliveries at home in the last ten years. In accordance with the data of the Turkey Demographic and Health Survey (TDHS), between 2008–2013 years, the percentage of unplanned home deliveries decreased by 8% to 3% (2, 3).

Prenatal care attendance is related to institutional deliveries (4). Women receiving prenatal care earlier and more frequently have more institutional deliveries (5). Prenatal care in early pregnancy could prevent congenital abnormalities, fetal growth restriction, prematurity or asphyxia (6). Furthermore, the use of prenatal care services, give health professionals an opportunity to inform women about their pregnancies and promote institutional delivery. Women advised on institutional delivery had 6.5 times more institutional births (7). The confidence between health care professionals and women plays an important role in following the advice on delivery place and it is established and strengthened by prenatal care (8).

In the United States of America, one in four women having unplanned deliveries at home has not received prenatal care (9). In India, unplanned home deliveries were reported three times more in women with ≤ 3 prenatal care visits (10). According to national data of our country, 20% of women who have never received prenatal care and only 0.8% of women received three or more prenatal care visits have unskilled deliveries (3). Prenatal care makes women familiar with health system and its facilities. The confounding factors in the use of prenatal care are knowledge level of women about pregnancy risks and complications, women’s attitudes toward health care, and other social factors (1).

Utilization of health services is affected not only accessibility but also demand for services which determined by largely socio-economic factors, personal beliefs, and perceptions (11). The relationship between prenatal care and health decisions were not fully understood. Poor quality and content of care and low level of women’s interaction with health care providers act as a barrier in front of institutional delivery (7,11). Additionally, dissatisfaction with rude, arrogant and neglectful behavior at health facilities result in unplanned home births (1).

Socio-economic and cultural factors are related to accessibility, the use, and quality of health care (12,13). The usage of antenatal care is highly affected by social and cultural status of population. By this time, the association between the content of prenatal care and institutional deliveries has not been studied in Turkey. The aim of this study was to compare institutional and unplanned home deliveries with regard to the use and content of prenatal care services.

## Materials and Methods

The research was designed as a case-control study and carried out in Istanbul which is the largest city in Turkey with a population of almost 14 million. In 2010, according to the data of the Public Health Directorate, 211088 live births and 229 unplanned home deliveries were reported. Two hundred and twenty-nine women unplanned home deliveries between 1 Jan-30 Jun 2011 and reported to the Public Health Directorate were involved in the study group and mothers having institutional births at the same time (two controls per case; n=458) were taken as control group. Eligible controls were determined and listed by the Family Medicine Information System and assigned via random number list. One hundred and seventy-three women (75.5%) in the study group and 386 (84.3%) in the control group could be reached.

After taking informed consent of all participants, the data was collected with a questionnaire by face to face interview technique. Participants were asked a series of questions in accordance with the national guidelines about prenatal care service they received during their recent pregnancy. Eight different procedures were evaluated (height and weight measurements, blood group determination, auscultation, complete blood count, urine analysis, blood pressure measurement, and ultrasonography). The data were analyzed using SPSS 20.0 software (Chicago, IL, USA). Frequencies, percentages, averages, and medians were calculated and Chi-square analysis was used in the evaluation of numerical data.

## Results

The socio- and bio-demographical characteristics of women are shown in Table 1. The socioeconomic and environmental factors of study and control groups and their relation with unplanned home deliveries were discussed detailed in another article (14). Six factors (presence of health insurance, lifetime in Istanbul, educational status of woman, household count, the age of woman at the time of current delivery, and the status of having received antenatal care) were determined as independent risk factors for unplanned deliveries at home. Among these, failure to receive antenatal care and educational status of woman seem to possess the highest risk (14).

**Table 1:** Socio- and bio-demographical features of women

***Variable***		***Home delivery***	***Intuitional delivery***	***P***
		n=173	n=386	
Husband’s work	Unemployed	68 (40.5)	78 (20.6)	0.001
	Employed	100 (59.5)	301 (79.4)	
Health insurance	Yes	68 (39,3)	52 (13.5)	0.001
	No	105 (60.7)	334 (86.5)	
Women work	Unemployed	6 (3.5)	24 (6.2)	0.180
	Employed	167 (96.5)	361 (93.8)	
Women education	Uneducated	92 (53.2)	59 (15.3)	0.001
	Primary school and above	81 (46.8)	326 (84.7)	
Husband’s education	Uneducated	32 (18.5)	18 (4.7)	0.001
	Primary school and above	141 (81.5)	367 (95.3)	
Age at birth	≤19 year	19 (11.0)	17 (4.4)	0.003
	≥20 year	154 (89.0)	369 (95.6)	
Pregnancy count	≥2	108 (62.4)	161 (41.8)	0.001
	1	33 (23.4)	110 (40.6)	
Unwanted pregnancy	Yes	53 (30.6)	67 (17.4)	0.001
	No	120 (69.4)	319 (82.6)	

Overall 16.2% (n=28) of the women with unplanned deliveries at home did not receive prenatal care. This rate was only 3.4% (n=13) in women with institutional deliveries (*P*=0.001). Majority of the women with unplanned home deliveries were given first prenatal care by family physicians (*P*=0.009) (Table 2). However, the content of prenatal care services was quite poor. All of the eight procedures mentioned above, except auscultation, were performed significantly less frequently in women delivered at home. The results are summarized in Table 3.

**Table 2:** Utilization of prenatal care in study and control groups

***Prenatal Care***		***Home delivery (n=173)***	***Institutional delivery (n=386)***	***P***
		n (%) ^ * ^	n (%) ^ * ^	
Source of prenatal care	Non-Received	28 (16.2)	13 (3.4)	0.001
	Family physician	7 (4.0)	8 (2.1)	
	Obstetrician	59 (34.1)	126 (32.6)	
	From both	80 (45.7)	239 (61.9)	
Physician of first prenatal care	Family physician	19 (13.1)	23 (6.2)	0.009
	Obstetrician	126 (86.9)	350 (93.8)	
Time of first prenatal care	Before 14 weeks	135 (93.1)	363 (97.6)	0.015
	After 15 weeks	10 (6.9)	9 (2.4)	

**Table 3:** Content of prenatal care in study and control groups

***Applied parameters***		***Home delivery n (%)***	***Institutional delivery n (%)***	***P***
Height measurement	Yes	48 (33.1)	161 (43.2)	0.036
	No	97 (66.9)	212 (56.8)	
Blood pressure measurement	Yes	131 (90.3)	357 (95.7)	0.019
	No	14 (9.7)	16 (4.3)	
Weight measurement	Yes	112 (77.2)	347 (93.0)	0.001
	No	33 (22.8)	26 (7.0)	
Auscultation	Yes	62 (42.8)	161 (43.2)	0.933
	No	84 (57.2)	212 (56.8)	
Complete Blood Count	Yes	113 (77.9)	356 (95.4)	0.001
	No	32 (22.1)	17 (4.6)	
Urine Analysis	Yes	101 (69.7)	343 (92.0)	0.001
	No	44 (30.3)	30 (8.0)	
Determining of Blood Groups	Yes	79 (54.5)	303 (81.2)	0.001
	No	66 (45.5)	70 (18.8)	
Ultrasonography	Yes	138 (95.2)	368 (98.7)	0.018
	No	7 (4.8)	5 (1.3)	

## Discussion

Prenatal care was hypothesized as having positive effects on promoting institutional deliveries. Besides, health workers have a chance to inform women about their status of pregnancy and risks by means of prenatal care services. We found that 16.3% of women had delivery at home did not receive prenatal care. Our results were compatible with national and international literature. The coverage rate of prenatal care was 97.0% in Turkey and 99.4% in Istanbul and 20% of women having delivery at home did not receive any prenatal care according to TDHS-2013 (3). About 81% of women throughout the world have received prenatal care at least once. This rate reached up to 95%–100% in developed countries whereas it was 22%–76% in underdeveloped countries (15). Institutional deliveries are more frequent in women given prenatal care service, especially 3 or more times (9,10,16– 19). We think two possible explanations for less frequently using of prenatal care service in home births. Firstly, these women could have lower familiarity with health care system due to their socio-demographic features.

Because receiving prenatal care was found as a marker of familiarity with the health system and its facilities (1). Receiving health information about prenatal care is strongly associated with institutional delivery (5). Secondly, the risk perceptions about pregnancy among these women could be different. The socio-cultural differences of these two groups may influence their perceptions and by that way, their decisions about seek care. Consistent with the cultural theory, people make selections and avoid risks depending on their social and individual circumstances (20). In the field of unplanned home births, women’s perceptions are mainly influenced by awareness of danger, individual past experiences with pregnancy and health care services.

An interesting finding of this study was the discrepancy of prenatal care content between women with different birthplaces. The content of these services was quite poor in women unplanned home births and this might be due to the impact of women’s socio-demographical characteristics on physicians’ behaviors. There is evidence that as a result of stereotypic beliefs, patients’ socio-demographic characteristics have an impact on both physicians’ behaviors, diagnoses, and treatments (21). Physicians’ decisions on treatment varied based on their perceptions about likeability and competence of simulated patients. Unlikeable patients have received less medical attention and follow-up care (22). We thought two possible explanations of this situation; if patients perceive that physicians like and take care of them, they are more volunteer to give information and be more active and satisfied in the encounter and more compliant with medical regimens (21). Another explanation of this result could be the perceived benefits of these services as mentioned before. The higher use of prenatal care and institutional delivery are linked to each other, usually the former results in the later (17). Institutional delivery was 4.59 times more in women with 1 or 2 prenatal care visit, and it was 17.92 times more in women with 3 or more prenatal care visits (11). Furthermore, women received more procedures of prenatal care were more likely to have institutional deliveries (18). A positive association was demonstrated between the quality of prenatal care and institutional delivery (19).

The most powerful aspect of our study is the collection of data in Istanbul, which is the largest metropolis in Europe that harbors one-fifth of the country population, within the next six months after birth. On the other hand, home-delivered women in our study were registered by the Public Health Directorate and it is possible that there could be non-registered women delivered at home. These constitute the main limitation of the study.

## Conclusion

This study demonstrates the association of birthplace with the use and content of prenatal care. In another word, the usage and adherence of prenatal care guidelines could increase the skilled institutional delivery. Therefore, policymakers should promote health professionals in compliance with prenatal care guidelines. Training health professionals in the field of prenatal care and patient communication could increase the quality of prenatal care and would promote institutional deliveries. However, our findings in this study do not demonstrate the whole picture, and there might be interactions between many other social factors as well.

## Ethical considerations

Ethical issues (Including plagiarism, informed consent, misconduct, data fabrication and/or falsification, double publication and/or submission, redundancy, etc.) have been completely observed by the authors.
